# Maternal nutritional status and milk volume and composition in India: an observational study

**DOI:** 10.1016/j.ajcnut.2023.02.002

**Published:** 2023-02-10

**Authors:** Melissa F. Young, Emily C. Faerber, Rukshan V. Mehta, Samriddhi Ranjan, Sweekruthi A. Shetty, Usha Ramakrishnan, Kannan Rangiah, Beena Bose, Sarita Devi, Pratibha Dwarkanath, Anura V. Kurpad, Sunita Taneja, Reynaldo Martorell

**Affiliations:** 1 Hubert Department of Global Health, Emory University, Atlanta, United States; 2 Centre for Global Child Health, The Hospital for Sick Children, Toronto, Canada; 3 Centre for Health Research and Development, Society for Applied Studies, New Delhi, India; 4 Food Safety and Analytical Quality Control Laboratory, CSIR-CFTRI, Karnataka, India; 5 Institute of Bioinformatics, Bangalore, India and Manipal Academy of Higher Education, Manipal; 6 Division of Nutrition, St. John's Research Institute, Bangalore, Karnataka, India; 7 Department of Physiology, St. John’s Medical College, Bengaluru, Karnataka, India

**Keywords:** maternal nutrition, body composition, human milk intake, breastfeeding, infant intake, India

## Abstract

**Background:**

Human milk provides essential nutrition for infants, and its benefits are well established. We lack data on the influence of maternal nutritional status on milk volume and composition in low-middle income countries.

**Objective:**

We aimed to *1)* assess lactation performance (human milk volume, macronutrient composition, and infant energy intake) in Indian females and *2)* examine the associations between maternal anthropometry (BMI, percentage body fat) and lactation performance.

**Methods:**

We conducted an observational study among 232 mother-infant dyads, 2 to 4 mo postpartum in Haryana, India. We used deuterium oxide dose-to-mother technique to measure milk volume and maternal percentage body fat and collected human milk samples to determine macronutrient and energy concentrations. Adjusted multiple linear regression models were used to examine the associations between maternal anthropometry and lactation performance.

**Results:**

The mean BMI and percentage body fat of mothers were 21.7 ± 3.6 kg/m^2^ and 29.5 ± 7.7, respectively. Milk volume and macronutrient composition were similar to the reference values (means ± standard deviations: milk volume, 724 ± 184 mL/d; median (25th, 75th percentile); protein, 9.9 (8.3, 11.7) g/L; fat, 41.0 ± 15.2 g/L; energy density, 0.71 ± 0.14 kcal/g; lactose, 65.5 (55.3, 71.3) g/L). Maternal BMI and percentage body fat were not significantly associated with macronutrient composition. Both maternal BMI and percentage body fat were negatively associated with milk volume (-7.0, 95% CI: -12.4, -1.6 mL/d; -3.5, 95% CI: -6.0, -1.1mL/d, respectively) but there were no effects on the total energy intake of infants after adjusting for covariates.

**Conclusion:**

Most mothers had a normal BMI and milk of similar composition and volume to reference values. Future work in populations with a greater burden of underweight and/or obesity are needed to examine the underlying mechanisms between maternal body composition and milk volume.

This trial was registered at The Clinical Trials Registry- India as CTRI/2017/01/007636.

## Introduction

Maternal and child malnutrition in India remain a public health challenge [1]. The use of growth curves based on data from breastfed infants [2] reveal a new pattern of early onset and severity of malnutrition (particularly in wasting) that the previous National Center for Health Statistics growth curves had masked [1, 3]. Although exclusive breastfeeding from birth to 6 mo remains one of the most cost-effective child survival interventions, little is known about how to prevent wasting in the exclusively breastfed child. For example, in Haryana, 25% and 18% of infants under 6 mo of age are wasted and stunted, respectively [4]. Mothers are often overlooked as an intervention route, yet their diets and nutritional reserves are the sole source of nourishment for infants, first *in utero* and then during the period of exclusive breastfeeding in the first 6 mo of life [5]. Despite some progress in reducing the prevalence of underweight among females in India (18.7%), the burden remains unacceptably high with large disparities noted by region and age [4, 6]. Furthermore, many low- and middle-income countries, including India, are increasingly facing the burden of malnutrition that has doubled with the rising prevalence of overweight and obesity among females of reproductive age (24%) [7].

The relationship between maternal nutrition and human milk composition is poorly understood. Recent meta-analyses by Leghi et al. [8] and Daniel et al. [9] report positive associations between maternal adiposity and milk fat concentration but no associations with energy density, total protein, or lactose concentrations. However, the authors express low confidence in their findings because of variability in study methodology and unexplained heterogeneity in outcomes of interest [8, 9]. Furthermore, low- and middle-income countries are underrepresented in the existing literature [9].

Still less is known about the relationship between maternal nutritional status and milk volume. These relationships are complex as there are biological, medical, and psychosocial factors that may contribute to suboptimal breastfeeding among females who are underweight, overweight, or obese [[10], [11], [12]]. Previous research has reported mixed results with maternal weight or postpartum weight changes and milk volume, with some reporting positive [13], negative [14], or null associations [15]. Estimating milk volume can be challenging and test weighing of infants before and after feeding episodes is a commonly used method; however, this method is prone to measurement errors, leading to underestimation of infant intake [[16], [17], [18]]. The deuterium oxide *dose-to-mother (DTM) technique* for assessing the total human milk intake overcomes many of these barriers to provide a more accurate depiction of average infant milk consumption over a 2-wk period, but fewer studies have used this approach [19].

Maternal malnutrition may be a key contributing factor to early growth failure in the Indian context; however, we lack the evidence base necessary for prioritizing mother-centered interventions during this period. Our overall hypothesis is that poor nutritional status is associated with poor lactation performance. We test this hypothesis in 2 ways *1)* comparing lactation performance (milk macronutrient composition, energy density, milk volume, and energy intake from milk) of Indian females at 2 to 4 mo postpartum to reference ranges; and *2)* examining the association of maternal nutritional status (maternal BMI and percentage body fat) with lactation performance.

## Methods

### Subjects

We recruited mother-infant dyads from a previously established pregnancy surveillance database from 3 primary health centers in peri-urban and urban regions of Faridabad district, Haryana, India. Dyads were eligible if they included a lactating mother aged 18 to 45 y, an infant 2 to 4 mo of age, and were likely to remain in the area for 2 wk after enrolment. Mothers who consumed guttka (beetel nut) or smoked tobacco at the time of the study were excluded. Trained data collectors visited the household to obtain written informed consent from at least 1 parent.

## Methods

### Data Collection and variable specification

Data collectors administered a questionnaire that assessed sociodemographic information; food security, using the *Household Food Insecurity Access Scale* [20]; maternal and infant morbidity; maternal diet; and breastfeeding behaviors. Data were collected from June 2017 to June 2018 to capture seasonality.

*Milk volume*. The deuterium oxide DTM technique was used to assess the total human milk intake, as specified by the *International Atomic Energy Agency* [19]. A 30-g dose of deuterium oxide (Product number: Q39316, Sercon, Cheshire, United Kingdom) was weighed via a calibrated analytical balance (Quintix 224-10IN, Sartorius, Goettingen, Germany) in the laboratory. Prepared doses were sealed with parafilm to prevent loss through evaporation and wrapped in an aluminum foil to conceal from light. Doses were stored at 4 to 8 °C until they were carried to the field in a labeled plastic chiller with temperature maintained at 4 to 8 °C.

Lactating mothers consumed a single, deuterium oxide dose on day 0, immediately followed by 15 mL of filtered/distilled water [19]. Saliva samples were collected from both mother and infant on day 0, before the administration of deuterium oxide, and then on days 1, 2, 3, 4, 13 and 14. Approximately 0.5 mL of saliva was collected at each time. A 30-min fast was maintained before collection of all saliva samples to ensure no residual food particles were present in the collected sample. Samples were stored in 2 mL acid washed cryogenic vials and sealed with parafilm strips to prevent loss of deuterium oxide through evaporation. Saliva samples were transported using a plastic chiller maintained with a temperature of 4 to 8 °C, then stored at -20 °C.

Deuterium enrichments of timed saliva samples from the mother and infant were measured by Fourier transform infrared spectroscopy (4500t FTIR, Agilent Technologies, Santa Clara, United States), and then used to estimate infants’ total human milk intake and non-milk water intake. Exclusive breastfeeding was determined based on a non-milk water intake <86.6 g/d [21] and by caregiver recall of the previous day [22]. The deuterium oxide DTM technique also provided an estimate of the mother's total body water from which the maternal body fat was calculated [19].

*Anthropometry.* Infant weight was measured to the nearest 20 g (Seca 385 infant scale, Seca GmbH & Co. KG, Hamburg, Germany). Maternal body weight and height were measured to the nearest 100 g (Seca 803) and nearest 1 mm (Seca 213), respectively. Secondary indicators of maternal nutritional status included: maternal mid-upper arm circumference (MUAC) and calf circumference, measured to the nearest 1 mm using a Seca 212 non-stretch, teflon tape, and maternal triceps and subscapular skinfold thickness were measured to the nearest 0.2 mm using a Holtain Tanner/Whitehouse caliper (610ND, Crosswell, United Kingdom). These measurements were recorded in duplicate on the day of enrolment.

*Milk macronutrient and energy content.* Milk samples were collected on day 1 after enrolment in the mothers’ homes. Mothers were asked not to breastfeed the baby for at least 1 h, then manually expressed milk from one breast completely. Samples were collected in a sterile acid washed, polypropylene specimen container (Genaxy, New Delhi, India), immediately chilled and stored at 2 to 8 °C for transport to the laboratory. At the laboratory, samples were centrifuged in a capillary tube in a *Creamatocrit Plus analyzer* (Separation Technology, Inc, Sanford, FL, USA) to estimate their fat concentration and calculate their energy density.

The remaining milk sample was aliquoted into a 10 mL falcon tube (acid washed, trace element free, centrifuge tube, Genaxy) and a 2 mL cryovial (virgin polypropylene, heavy metal, and trace element free, Genaxy), for storage at -80 °C for protein and lactose concentrations.

Total protein content was estimated by bicinchoninic acid assay kit [23]. All samples were analyzed in triplicate for protein estimation. Lactose concentration was estimated using Liquid Chromatography-Mass Spectrometry Agilent 1290 infinity II UHPLC system (Agilent Technologies India Pvt. Ltd., India) connected with Sciex 6500 QTRAP (Sciex, Singapore), in triplicate using standard protocols [23].

### Data analyses

All continuous variables were assessed for normality based on the visual inspection of histograms and measures of skewness. Skewed outcome variables were log transformed. Basic summary statistics are presented as mean ± SD for normally distributed variables; median (25th percentile, 75th percentile) for skewed variables; and (n) % for categorical variables.

To assess differences in milk composition, volume, and infant energy intake from human milk by maternal BMI category (underweight BMI, <18.5 kg/m^2^; normal weight BMI, 18.5–24.9 kg/m^2^; and overweight/obese BMI, ≥25.0 kg/m^2^) we used analysis of variance (for normally distributed outcome variables) and Kruskal-Wallis test (for skewed outcome variables).

We used multiple linear regression models to assess the relationships between maternal anthropometry and lactation performance and report results of 3 models: model 1 is unadjusted; model 2 is adjusted for maternal age, infant age, and infant sex; model 3 is also adjusted for additional sources of variation, namely infant weight-for-age z-score (WAZ), infant morbidity (caregiver-reported fever, diarrhea, and/or difficulty breathing in the previous 2 wk), and exclusive breastfeeding in the previous day. Covariates were selected *a priori* based on the theoretical evidence on potential important confounders of the relationship between maternal nutrition and lactation performance. Protein and lactose concentrations were both right skewed and were thus log transformed for regression modeling. We assessed model assumptions by visual inspection of residual plots and Cook’s D and evaluated multicollinearity with variance inflation factors. We also stratified exclusive breastfeeding status to assess the potential effect modification.

To enable interpretability and comparisons among anthropometric indicators and outcomes within this study [24], all maternal anthropometric indicators and outcome variables were standardized by subtracting the sample mean from each observed value and dividing by the sample SD. We then used these standardized values to re-run regression models to yield coefficients (β_stand_) that represent the change, in SDs, in the outcome per one SD change in exposure. Sample size of 232 mother-infant dyads was 80% powered to detect a 0.2 correlation between exposure and outcome (alpha < 0.05), and assuming a 10% loss to follow-up over the course of data collection. No imputations were made for the missing data (<10% missing in dataset). Final analytic sample size included in Supplemental Figure 1. All analyses were completed in SAS 9.4 (SAS Institute, Cary, NC, USA).

### Ethical considerations

Ethical approval for this study was obtained by the ethics committee of *the Society of Applied Studies* and by *the Emory University Institutional Review Board*. Approval was also obtained from the *State government and Health Ministry’s Screening Committee of the Indian Council of Medical Research*. This observational study is registered with Clinical Trials Registry - India, CTRI/2017/01/007636.

## Results

Demographic characteristics of the 232 participating dyads are provided in Table 1. Mothers’ mean age was 24.7 ± 3.9 y, and 77.4% of households were classified as food secure. The mean maternal BMI was 21.7 ± 3.6 kg/m^2^, and percentage body fat was 29.5 ± 7.7. Most mothers (69.0%) were classified as normal weight by BMI; 14.7% and 16.4% were classified as underweight and overweight/obese, respectively. The mean age of infants was 3.0 ± 0.6 mo, and 20.0% were classified as stunted, 8.2% as wasted and <1% were overweight or obese. Over half (56.8%) of the infants were exclusively breastfed based on maternal recall and this increased to 67.8% when calculated based on the DTM data. Of the infants who were non-exclusively breastfeed, only 3 reported consuming solid, semi-solid, or soft food, and the remaining infants were primarily provided water, animal milk, tea or coffee, or infant formula in addition to the human milk.

**TABLE 1:** Characteristics of lactating females and infants 2 to 4 mo postpartum in Haryana, India (*n* = 232).^1^LAZ, length for age z-score; WAZ, weight for age z-score; WLZ and WFL, weight for length z-score.

**Household demographic characteristics**	
Maternal age, y	24.7 ± 3.9
Maternal education, y	7.3 ± 4.9
Maternal occupation, housewife, % (n)	97.0 (225)
Number of children	2.3 ± 1.2
Below poverty line card, % (n)	22.8 (53)
Religion, % (n)	
Hindu	70.7 (164)
Muslim	28.0 (65)
Other	1.3 (3)
Caste, % (n)	
Scheduled caste	22.8 (53)
Scheduled tribe	0.4 (1)
Other backward caste	49.1 (114)
Other caste	27.6 (64)
Food secure^2^, % (n)	77.4 (178)
**Maternal anthropometry**	
Weight, kg	50.2 ± 9.2
Height, cm	152.1 ± 6.1
BMI, kg/m^2^	21.7 ± 3.6
Underweight, BMI < 18.5, % (n)	14.7 (34)
Normal weight, BMI 18.5–24.9, % (n)	69.0 (160)
Overweight, BMI 25 - 29.9, % (n)	13.8 (32)
Obese, BMI > 30, % (n)	2.6 (6)
Percentage body fat	29.5 ± 7.7
MUAC, cm	25.3 ± 3.4
Calf circumference, cm	31.1 ± 3.6
Triceps skinfold, mm	16.1 ± 6.4
Subscapular skinfold, mm	14.8 ± 5.7
**Child characteristics, %(n)**	
Sex, F	47.4 (110)
Age, mo	3.0 ± 0.6
Birth weight, kg^4^	2.8 ± 0.5
Low birth weight, % (n)	18.3 (40)
Stunted (LAZ < - 2 SD)	20.0 (46)
Underweight (WAZ < - 2 SD)	19.5 (45)
Wasted (WLZ < -2 SD)	8.2 (19)
Overweight (BMI > 2 SD)	1 (0.43)
Overweight (WFL > 2 SD)	2 (0.86)
Obese (BMI > 3 SD; WFL > 3 SD)	1 (0.43)
Any recent child morbidity^3^	31.2 (72)
**Child feeding practices, %(n)**	
Prelacteal fed	23.3 (54)
Exclusively breastfed (maternal recall)^5^	56.8 (130)
Exclusively breastfed (dose-to-mother)^6^	67.8 (156)

1 All values are mean ± standard deviation unless otherwise specified [% (n)].

2 Measured via the Household Food Insecurity Access Scale.

3 Caregiver-reported fever, diarrhea, or difficulty breathing within the last 2 wk.

4 Source of birth weight was either the immunization card or birth certificate (18.5%), recall (52.2%), or other record (23.7%) and was missing for 5.6% of respondents.

5 Based on maternal recall of the previous day [22].

6 Based on estimated non-milk water intake of less than 86.6 grams per day [21] as determined by the deuterium oxide dose-to mother technique [19].

Mean milk fat concentration was 41.0 ± 15.2 g/L, which is similar to the reference values provided by the *Institute of Medicine* [25] and to those reported in a recent systematic review by Leghi et al. [26] (Table 2). The median (25th percentile, 75th percentile) concentrations of protein and lactose were 9.9 g/L (8.3, 11.7) and 65.5 g/L (55.3, 71.3), respectively, and similar to reference values reported [25, 26]. The mean energy density was 0.71 ± 0.14 kcal/g, which is somewhat higher than the values cited by the *World Health Organization* in developing countries, but in alignment with the values cited for industrialized countries [27]. Mean milk volume for all infants (exclusively and non-exclusively breastfed) was 724 ± 184 mL/d, just below the reference range of approximately 728 to 777 mL/d as reported in industrialized countries among exclusively breastfed infants in the first 4 to 5 mo of life [25]. As described in Table 3, milk volume was higher for exclusively breastfed infants (776 ± 146 mL/d) than for non-exclusively breastfed infants (660 ± 208 mL/d) and in non-wasted (734 mL/d) than in wasted infants (602 mL/d). Infants had a mean intake of 527 ± 150 kcal/d (Table 2), which is slightly below the reference range of 537 to 608 kcal/d [28]. However, the energy intakes for exclusively breastfed infants (568 kcal/d) were within reference ranges, whereas non-exclusively breastfed infants had notably low energy intakes from human milk (474 kcal/d). Infant energy intake was also lower among wasted (446 kcal/d) than non-wasted (533 kcal/d) infants. None of these outcomes differed between underweight, normal weight, and overweight/obese mothers (*P* > 0.05). Maternal BMI categories were likewise not associated with the infant size (Supplemental Table 1).

**TABLE 2:** Human milk macronutrient composition and volume among lactating females 2 to 4 mo postpartum in Haryana, India (*n* = 232)^1^

	Mean ± SD or Median (Q1, Q3)	Reference values [25, 26]	
		*Institute of Medicine*, 1991	Leghi, 2020^2^
Fat concentration, g/L	41.0 ± 15.2	39.0 ± 4.0	42.4 ± 13.1
Protein concentration, g/L	9.9 (8.3, 11.7)	10.5 ± 2.0	13.3 ± 4
Lactose concentration, g/L	65.5 (55.3, 71.3)	72.0 ± 2.5	64.3 ± 8.6
Milk volume, mL/d	724 ± 184	728–777^3^	
Energy density, kcal/g	0.71 ± 0.14	0.53–0.70; 0.60–0.83^4^	
Energy intake, kcal/d	527 ± 150	537–608^5^	

1 Values are means ± SD or median (25th percentile, 75th percentile). Note 5 participants are missing values for fat concentration/energy density, 1 participant is missing data for milk volume, and 6 for energy intake. Q1, 25^th^ percentile; Q3, 75^th^ percentile.

2 Values are reported for “full expression” samples collected in the morning.

3 Reported average intake in industrialized countries “in the first 4 to 5 mo” is 750–800 g/d [25]; converted to mL/d assuming a density of 1.03 g/mL.

4 As reported by the WHO in 1998, based on a review of literature, energy density of human milk “ranged from 0.53 to 0.70 kcal/g in developing countries and from 0.60 to 0.83 kcal/g in industrialized countries” [27].

5 According to “Human energy requirements,” a report of a Joint FAO/WHO/UNU Expert Consultation, 2001; range of daily energy requirements for male and female infants 2 to 4 [28].

**TABLE 3:** Milk composition and intake by select infant and household factors in Haryana, India (*n***=** 232) 1^,^∗

		n	Fat concentration, g/L	Protein concentration, g/L	Lactose concentration, g/L	Energy density, kcal/g	Milk volume, mL/d	Energy intake, kcal/d
Infant sex	Male	122	41.3 ± 15.9	9.9 (8.5, 11.5)	65.8 (55.0, 70.5)	0.71 ± 0.14	750 ± 187∗	548 ± 151∗
	Female	110	40.7 ± 14.5	9.8 (8.2, 12.0)	65.5 (56.5, 72.5)	0.71 ± 0.13	696 ± 176	504 ± 146
Exclusively breastfed (maternal recall)^2^	Yes	130	41.3 ± 15.1	9.9 (8.4, 11.7)	65.8 (58.0, 72.0)	0.72 ± 0.14	776 ± 146∗	568 ± 134∗
	No	99	40.9 ± 15.5	9.8 (8.1, 12.0)	65.0 (52.5, 71.0)	0.71 ± 0.14	660 ± 208	474 ± 154
Exclusively breastfed (dose-to-mother)^3^	Yes	156	39.7 ± 14.6∗	9.6 (8.2, 10.9)∗	65.5 (57.5, 71.3)	0.70 ± 0.13∗	771 ± 155∗	554 ± 137∗
	No	74	44.4 ± 16.0	11.2 (8.5, 12.9)	66.3 (53.0, 72.5)	0.74 ± 0.15	626 ± 201	470 ± 160
Recent infant illness	Yes	72	39.8 ± 13.5	10.3 (8.2, 12.5)	67.5 (57.5, 74.3)∗	0.70 ± 0.12	721 ± 202	518 ± 168
	No	159	41.7 ± 15.8	9.7 (8.3, 11.3)	64.5 (55.0, 70.5)	0.72 ± 0.14	726 ± 176	532 ± 141
Socioeconomic tertile	Lowest	77	38.8 ± 14.2	9.7 (8.1, 11.4)	67.5 (53.0, 74.0)	0.69 ± 0.13	705 ± 170	500 ± 138
	Mid	78	42.2 ± 14.7	10.1 (8.8, 12.1)	64.0 (56.5, 70.5)	0.72 ± 0.13	750 ± 199	552 ± 160
	Highest	77	42.1 ± 16.5	9.8 (8.1, 11.6)	66.5 (56.5, 70.5)	0.72 ± 0.15	717 ± 180	527 ± 148
Infant nutritional status	Stunted	46	42.3 ± 15.5	9.6 (8.4, 11.9)	65.5 (59.0, 70.5)	0.72 ± 0.14	684 ± 155	506 ± 132
	Not stunted	184	40.8 ± 15.2	10.0 (8.3, 11.7)	66.0 (55.0, 71.8)	0.71 ± 0.14	737 ± 186	534 ± 152
	Wasted	19	42.6 ± 18.6	9.8 (7.2, 11.1)	67.5 (58.0, 71.0)	0.73 ± 0.17	602 ± 219∗	446 ± 176∗
	Not wasted	212	40.8 ± 14.9	9.9 (8.3, 11.8)	65.5 (55.0, 71.5)	0.71 ± 0.14	734 ± 176	533 ± 145
Infant age	< 3 mo	112	41.1 ± 15.1	10.3 (9.0, 12.1)∗	67.5 (58.0, 72.5)	0.71 ± 0.14	714 ± 182	520 ± 149
	≥ 3 mo	120	40.9 ± 15.4	9.5 (7.9, 11.1)	64.0 (54.3, 70.5)	0.71 ± 0.14	734 ± 186	532 ± 151
Maternal weight status	Underweight	34	39.5 ± 15.7	9.8 (8.2, 11.0)	67.0 (52.5, 72.5)	0.70 ± 0.14	756 ± 182	539 ± 146
	Normal weight	160	41.1 ± 14.5	9.8 (8.4, 12.0)	66.0 (56.3, 71.8)	0.71 ± 0.13	717 ± 189	523 ± 157
	Overweight/obese	38	42.3 ± 17.7	10.0 (7.9, 11.8)	63.5 (55.0, 70.0)	0.72 ± 0.16	726 ± 163	529 ± 120

*∗ P* < 0.05 using ANOVA for normally distributed outcome variables and Kruskal-Wallis test for non-normally distributed outcome variables.

1 Values are means ± SD or median (25th percentile, 75th percentile). Note 5 participants are missing values for fat concentration/energy density, 1 participant is missing data for milk volume, and 6 for energy intake.

2 Based on maternal recall per the *WHO* indicator for exclusive breastfeeding [22].

3 Based on the deuterium oxide dose-to-mother technique [19], using a cutoff of 86.6 g/d of non-milk water intake [21].

**FIGURE 1:**
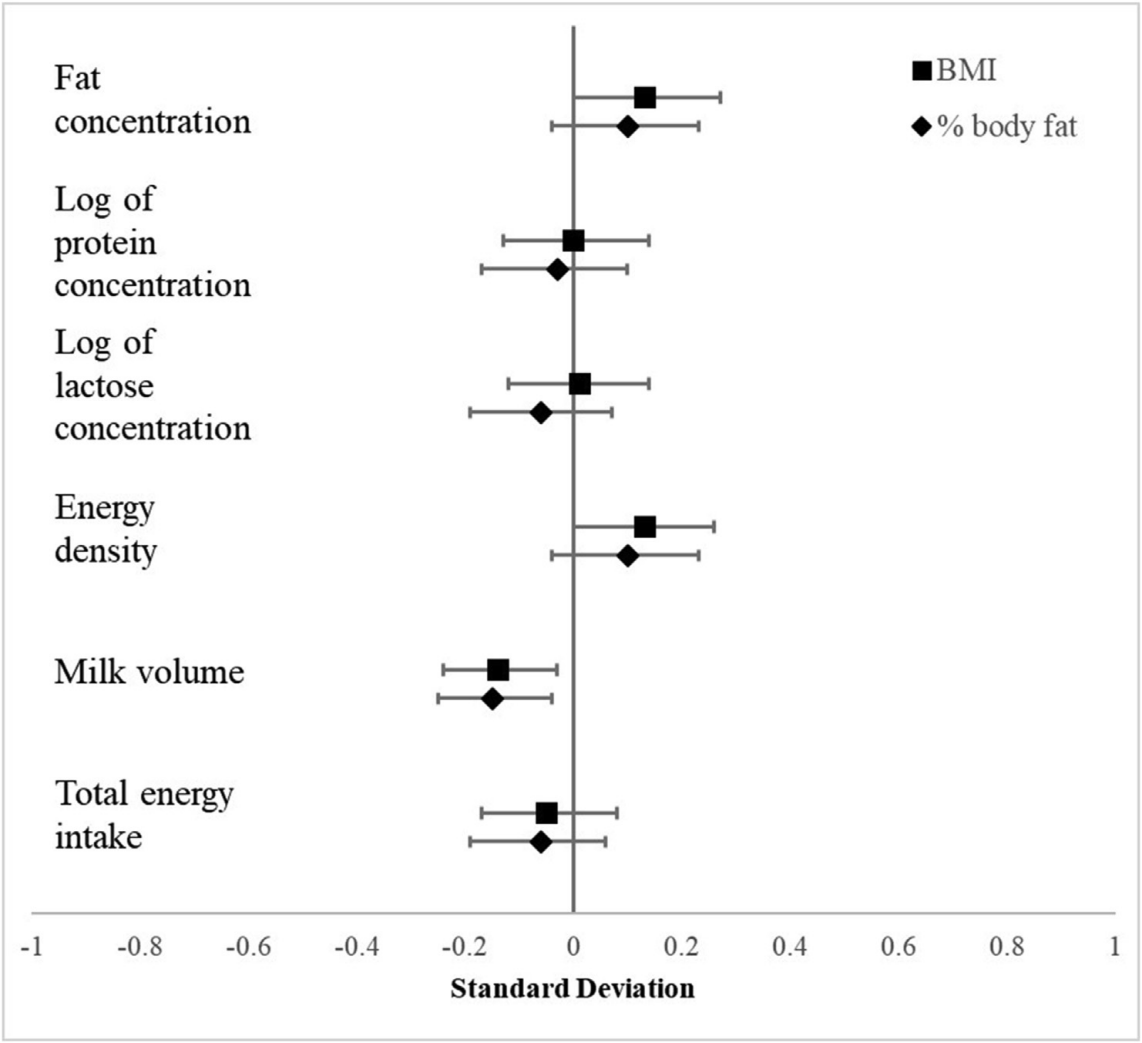
Association of maternal BMI and percentage body fat with human milk macronutrient composition and infant intake among lactating females 2 to 4 mo postpartum (*n =* 232). Results of linear regression models where anthropometric indicators and outcome variables have been standardized; coefficients therefore represent the change in standard deviations of the outcome variable with a one standard deviation change in anthropometric indicator. Error bars show 95% CI. Models are adjusted for maternal age; whether the infant was reported ill in the previous 2 wk; child age, sex, and weight-for-age z-score; and whether the infant was exclusively breastfed in the previous day.

Associations between maternal BMI and percentage body fat and lactation performance are presented in Table 4 for unstandardized coefficients and Figure 1 for standardized coefficients. Supplemental Table 2 and Supplemental Figure 2 show results for all 7 secondary maternal anthropometric indicators. Maternal BMI was positively associated with milk fat concentration in the partially adjusted model (β = 0.58; 95% CI: 0.00, 1.16), although relationships were not significant in the fully adjusted model (β = 0.56; 95% CI: -0.02, 1.13). Results for energy density mimic results for milk fat concentration and significant associations were only significant for the partially adjusted model (β = 5.31; 95% CI: 0.05, 10.57) but not the fully adjusted model (β = 5.08; 95% CI: -0.14, 10.30), although the effect estimates remained similar. There were no associations between maternal BMI and milk protein or lactose concentrations. Maternal percentage body fat was not associated with macronutrient composition.

**TABLE 4:** Results of multiple linear regression modeling human milk composition and volume and among maternal-infant dyads in Haryana, India (*n***=** 232).^1^^,^∗

	Fat concentration (g/L)	Log of protein concentration (g/L)	Log of lactose concentration (g/L)	Energy density (kcal/kg)	Milk volume (mL/d)	Energy intake (kcal/d)
**Model 1: Unadjusted**						
Maternal BMI	0.48 (-0.08, 1.03)	-0.06 (-1.04, 0.94)	-0.27 (-1.09, 0.56)	4.38 (-0.70, 9.46)	-4.7 (-11.4, 1.9)	-1.1 (-6.6, 4.4)
Percentage body fat	0.19 (-0.08, 0.45)	-0.12 (-0.58, 0.35)	-0.27 (-0.65, 0.12)	1.71 (-0.68, 4.10)	-3.0 (-6.1, 0.1)	-1.2 (-3.8, 1.4)
**Model 2: Adjusted for maternal age, infant age, infant sex**						
Maternal BMI	0.58 (0.00, 1.16)∗	0.10 (-0.92, 1.12)	-0.01 (-0.85, 0.85)	5.31 (0.05, 10.57)∗	-5.7 (-12.5, 1.1)	-1.0 (-6.7, 4.6)
Percentage body fat	0.21 (-0.05, 0.48)	-0.07 (-0.55, 0.40)	-0.19 (-0.58, 0.20)	1.97 (-0.47, 4.41)	-3.0 (-6.1, 0.2)	-0.9 (-3.5, 1.7)
**Model 3: Adjusted for maternal age, infant age, infant sex, recent illness, exclusive breastfeeding determined by dose-to-mother**^2^**, infant weight-for-age**						
Maternal BMI	0.56 (-0.02, 1.13)	0.01 (-0.99, 1.02)	0.06 (-0.79, 0.91)	5.08 (-0.14, 10.30)	-7.0 (-12.4, -1.6)∗	-1.9 (-7.1, 3.3)
Percentage body fat	0.19 (-0.07, 0.46)	-0.12 (-0.58, 0.35)	-0.19 (-0.58, 0.21)	1.78 (-0.65, 4.22)	-3.5 (-6.0, -1.1)∗	-1.3 (-3.7, 1.1)

*∗ P* < 0.05.

1 Note 5 participants are missing values for fat concentration/energy density, 1 participant is missing data for milk volume, and 6 for energy intake.

2 Exclusive breastfeeding defined as having <86.6 g/d of non-milk water intake using the deuterium oxide dose-to-mother technique [19, 21].

Maternal BMI (β = -7.0; 95% CI: -12.4, -1.6) and percentage body fat (β = -3.5, 95% CI: -6.0, -1.1) were negatively associated with milk volume (Table 4). Although the direction of associations was consistent across unadjusted and partially and fully adjusted models; the strongest and significant associations were noted in the fully adjusted model (adjusting for maternal age, infant age, infant sex, infant WAZ and morbidity, and exclusive breastfeeding). Total energy intake was not significantly associated with maternal BMI or percentage body fat. In the fully adjusted model, 5 of 7 anthropometric indicators were negatively associated with milk volume (β_stand_: -0.12 to -0.20). Only triceps and subscapular skinfold thickness showed no association with milk volume (Supplemental Table 2).

When stratified by exclusive breastfeeding, some differences were noted, although not consistently across anthropometric indicators for any given outcome (Supplemental Figure 3). The negative associations between maternal BMI and percentage body fat and milk volume were significant only among exclusively breastfeed infants.

## Discussion

We find little evidence that poor maternal nutritional status is associated with poor lactation performance between 2 and 4 mo postpartum in our study of Indian females. Most lactating mothers in our sample had a normal BMI and produced milk with macronutrient concentrations and energy density consistent with the reference ranges [[25], [26], [27]]. Total milk volume and energy intake were just below the lower ranges, although the mean intakes of exclusively breastfed infants were within reference ranges. We used references published by the *Institute of Medicine* [25] and results of a recent systematic review by Leghi et al. [26] of human milk macronutrient composition for the purpose of comparing our population to previously reported values. The values reported by Leghi et al. [26] are within ranges reported in a recent review of milk composition of lactating females in the United States and Canada [29] and India [39] that found variability in macronutrient concentrations and overall limited data on milk composition. However, these values are outdated and rely on studies that use inconsistent methods for both milk sample collection and biochemical analysis. The development of the standards for human milk composition is an area of active research [30].

In our study, maternal BMI and percentage body fat were not significantly associated with human milk macronutrient composition in the fully adjusted models. Across different maternal nutrition anthropometric indicators, there was some heterogeneity in the magnitude and significance of association; however, these findings which are consistent with previously reported results of recent reviews. Daniel et al. [9] found that a 1 kg/m^2^ increase in maternal BMI was associated with a 0.56 g/L (95% CI: 0.34, 1.1) increase in fat concentration; this is similar in both direction and magnitude to the finding reported here for maternal BMI and fat concentration, although the significance varied based on covariates included in model (partially adjusted model [β = 0.58 (0.00, 1.16): fully adjusted models (β = 0.56; 95% CI, -0.02, 1.13)]. In a meta-analysis by Leghi et al., mature milk fat concentration in females who were overweight/obese averaged [2.73 g/L (95% CI: 0.57, 4.89)] higher than milk fat concentrations among females who were normal weight [8]. Although we found no unadjusted differences in milk composition by BMI categories, only 16.4% of our sample was classified as overweight/obese.

Milk fat is highly correlated with energy density [31]. We observed nearly identical standardized coefficients between maternal BMI and energy density as were observed for fat concentration. Daniel et al. [9] reported a non-significant relationship between maternal BMI and energy density, with a β = 3.9 kcal/L (95% CI: -1.6, 9.5), which is similar to our non-standardized β = 5.08 kcal/kg (95% CI: -0.14, 10.30) for maternal BMI.

We observed an inverse relationship between several measures of maternal anthropometry and milk volume, that increased in strength after adjusting for additional sources of variation in milk volume, namely infant weight-for-age. Infant weight is typically correlated with both maternal weight and milk volume, although in our sample we only noted associations with milk volume. The relationship between infant weight and milk volume is complex and could be bidirectional. Infant weight can influence the *demand* for milk volume (bigger babies have greater nutritional requirements and consume more milk). On the other hand, mothers who have greater milk *supply* can in turn enable faster infant growth. Infant weight could also confound the relationship between maternal adiposity and milk volume, and including this covariate could isolate the direct relationship of maternal nutrition status and milk volume. It is also possible that our final model overcontrolled for covariates. Thus, we provided unadjusted and basic adjustments for comparison. Previous research has also reported an inverse relationship between maternal adiposity and milk volume. Diana et al. [14] observed that, among Indonesian mothers, a 1% increase in maternal body fat was associated with a -4.9 mL (95% CI: -9.6, -0.2) decrease in milk volume. Likewise we report a coefficient of -4.9 (95% CI: -7.5, -2.3) between maternal percentage body fat and milk volume. Possible mechanisms that may explain this inverse relationship are unclear. Endocrine and metabolic dysfunction, systemic inflammation, altered mammary gland development, delayed lactogenesis, and psychosocial factors have been discussed elsewhere as mechanisms to explain low rates of breastfeeding observed among females who are overweight and obese [10, 32]. It is notable that we see these trends even in a population of largely normal weight participants.

An alternative explanation could be that infants regulate their intake based on the energy density of human milk. Our results suggest that the inverse relationship between maternal anthropometry and infant energy intake is attenuated compared with the inverse relationship between maternal anthropometry and milk volume—as evidenced by the smaller standardized regression coefficients. Fomon et al. [33] first noted that infants may regulate intake in 1975, when it was observed that infants consumed greater quantities of low energy formula. In breastfed infants, it has been observed that high fat concentration and energy density are positively associated with measures of infant satiety at 1 and 3 mo [34], as well as low overall infant intake [[35], [36], [37], [38]]. Thus, infant regulation of milk intake could be another possible mechanism to explain low total milk volume with increasing maternal adiposity observed in our population. These relationships merit further investigation.

There are important limitations of this research. The cross-sectional design does not allow us to establish causality or eliminate potential biases. We cannot rule out reverse causality between associations reported in this article. Future research with a prospective study design would be ideal for better understanding the complex and inter-connected associations between maternal nutrition, milk composition, and infant growth. Furthermore, although we had several indicators of maternal anthropometry (weight, height, MUAC, calf circumference, and skin folds), a one-time anthropometric assessment of lactating females could fail to capture changes in body composition that occur across the spectrum of preconception, pregnancy, and postpartum and may influence lactation performance. Further research and validation work are needed to develop optimal nutrition indicators during these critical and dynamic physiological stages. These analyses also do not account for maternal physical activity or diet. An important strength of this research, however, is our ability to assess milk volume by the *deuterium oxide DTM technique*, which is more accurate and less disruptive than test weighing and also provides an estimate of usual intake across a 2-wk period [19]. We present both unstandardized and standardized coefficients, which enable comparison of the relationships between outcomes and between multiple anthropometric indicators. Our findings may not be generalizable to other populations with a greater burden of maternal underweight and/or obesity as most participants in our study had normal BMI. In contrast, 20% of infants were stunted, 8% were wasted and <1% were obese. Further research into the determinants of early growth faltering in this context is needed.

In conclusion, in our population of primarily normal weight mothers in India, milk composition and volume were generally within ranges of reported reference values. Relationships between maternal nutrition and lactation performance are complex, and both over and undernutrition may adversely impact milk composition and volume [[10], [11], [12]]; however, overall maternal nutrition had a minimal impact on total infant intakes. Total caloric intake of the infants was not influenced by maternal nutritional status in our population. However, it is important to examine this further in populations with a greater double burden of malnutrition to better understand the dual role that maternal over and undernutrition may play on lactation performance and infant growth.

We would like to kindly thank the data collection teams for their important contributions to the project and research fellow, Ms. Roshni M Pasanna (St. John's Research Institute, Bangalore) for her assistance in saliva sample analysis by Fourier transform infrared spectroscopy (FTIR). We are grateful to Jiangda Ou for technical assistance with the data and preliminary work. Our team greatly appreciates the time and collaboration of all participants that made this work possible.

MFY,UR, KR, AVK, ST, and RM designed the research; SR, SAS, KR, BB, SD, PD, AVK, and ST performed data collection/sample analysis and MFY, ECF, and RVM the analyzed data; MFY, ECF wrote the article; all authors (MFY, ECF, RVM, SR, SAS, UR, KR, BB, SD, PD, AVK, ST, and RM) critically read and provided feedback on manuscript, MFY had the primary responsibility for final content. All authors read and approved the final manuscript. The authors report no conflicts of interest.

## Data availability

Data described in the manuscript, code book, and analytic code will be made available upon request pending approval of research teams.

## Appendix A Supplementary data

Multimedia component1 Multimedia component1
