# Supplementary material for: Maternal nutritional status and milk volume and composition in India: an observational study
Source: Am J Clin Nutr. 2023 Feb 10;117(4):830–7. doi: 10.1016/j.ajcnut.2023.02.002 (PMC10273082; doi:10.1016/j.ajcnut.2023.02.002)
Supplement: Multimedia component1 [file mmc1.docx]

**Online Supporting Material**

**Maternal nutritional status and milk volume and composition in Indian females: observational study**

Melissa F. Young, Emily C. Faerber, Rukshan V. Mehta, Samriddhi Ranjan, Sweekruthi A Shetty, Usha Ramakrishnan, Kannan Rangiah, Beena Bose, Sarita Devi, Pratibha Dwarkanath, Anura V. Kurpad, Sunita Taneja, Reynaldo Martorell

**
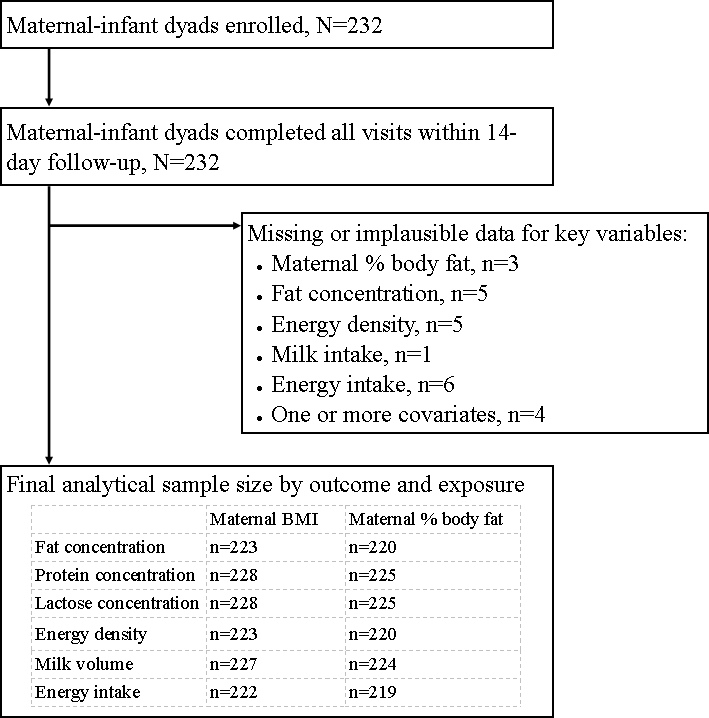
**

**Supplemental Figure 1.** Participant flow chart.

**Online Supporting Material**

| **Supplemental Table 1: Maternal BMI categories and size of infant** | | | | |
| --- | --- | --- | --- | --- |
|  | Underweight | Normal | Overweight/Obese |  |
| Z-scores | (Mean ± SD) | (Mean ± SD) | (Mean ± SD) | p-value |
| Weight-for-age: | -1.17 ± 0.85 | -1.26 ± 1.12 | -0.93 ± 0.87 | 0.22 |
| Length-for-age | -1.09 ± 1.43 | -1.01 ± 1.15 | -0.60 ± 1.08 | 0.12 |
| Weight-for-length: | -0.33 ± 1.37 | -0.58 ± 1.03 | -0.60 ± 0.88 | 0.44 |

**Online Supporting Material**

**Supplemental Table 2. Results of multiple linear regression modeling human milk composition and volume for multiple indicators of anthropometry among maternal-infant pairs in Haryana, India (n=232).^1,2^**

|  | **Fat Concentration (g/L)** | **Log of protein concentration (g/L)** | **Log of lactose concentration (g/L)** | **Energy density (kcal/kg)** | **Milk volume (mL/d)** | **Energy Intake (kcal/d)** |
| --- | --- | --- | --- | --- | --- | --- |
| **Model 1: Unadjusted** | | | | | | |
| Maternal BMI | 0.48 (-0.08, 1.03) | -0.06 (-1.04, 0.94) | -0.27 (-1.09, 0.56) | 4.38 (-0.70, 9.46) | -4.7 (-11.4, 1.9) | -1.1 (-6.6, 4.4) |
| Percent body fat | 0.19 (-0.08, 0.45) | -0.12 (-0.58, 0.35) | -0.27 (-0.65, 0.12) | 1.71 (-0.68, 4.10) | -3.0 (-6.1, 0.1) | -1.2 (-3.8, 1.4) |
| MUAC | 0.32 (-0.27, 0.92) | -0.44 (-1.48, 0.61) | -0.37 (-1.24, 0.52) | 2.95 (-2.48, 8.38) | -2.3 (-9.5, 5.0) | 0.6 (-5.4, 6.6) |
| Calf circumference | 0.01 (-0.56, 0.57) | 0.17 (-0.82, 1.17) | -0.04 (-0.87, 0.79) | 0.07 (-5.10, 5.23) | -4.1 (-10.9, 2.7) | -3.1 (-8.7, 2.5) |
| Triceps skinfold thickness | 0.15 (-0.16, 0.46) | -0.07 (-0.62, 0.48) | -0.22 (-0.68, 0.25) | 1.37 (-1.47, 4.22) | 1.3 (-2.4, 5.1) | 1.9 (-1.2, 5.0) |
| Subscapular skinfold thickness | 0.33 (-0.01, 0.68) | 0.24 (-0.38, 0.85) | -0.20 (-0.71, 0.32) | 3.03 (-0.15, 6.22) | -0.8 (-5.0, 3.3) | 1.5 (-1.9, 5.0) |
| Height | -0.06 (-0.39, 0.26) | 0.47 (-0.11, 1.05) | 0.37 (-0.11, 0.86) | -0.58 (-3.56, 2.41) | -0.3 (-4.2, 3.7) | -0.1 (-3.3, 3.1) |
| **Model 2: Adjusted for maternal age, infant age, infant sex** | | | | | | |
| Maternal BMI | 0.58 (0.00, 1.16)* | 0.10 (-0.92, 1.12) | -0.01 (-0.85, 0.85) | 5.31 (0.05, 10.57)* | -5.7 (-12.5, 1.1) | -1.0 (-6.7, 4.6) |
| Percent body fat | 0.21 (-0.05, 0.48) | -0.07 (-0.55, 0.40) | -0.19 (-0.58, 0.20) | 1.97 (-0.47, 4.41) | -3.0 (-6.1, 0.2) | -0.9 (-3.5, 1.7) |
| MUAC | 0.43 (-0.19, 1.06) | -0.26 (-1.35, 0.85) | -0.06 (-0.97, 0.86) | 3.96 (-1.73, 9.65) | -3.7 (-11.1, 3.8) | 0.5 (-5.7, 6.7) |
| Calf circumference | 0.07 (-0.51, 0.66) | 0.35 (-0.67, 1.38) | 0.23 (-0.62, 1.09) | 0.69 (-4.70, 6.07) | -5.5 (-12.4, 1.4) | -3.5 (-9.3, 2.3) |
| Triceps skinfold thickness | 0.19 (-0.13, 0.50) | -0.00 (-0.57, 0.56) | -0.11 (-0.58, 0.36) | 1.68 (-1.22, 4.59) | 1.2 (-2.6, 5.0) | 2.1 (-1.0, 5.2) |
| Subscapular skinfold thickness | 0.36 (0.01, 0.71)* | 0.29 (-0.33, 0.92) | -0.13 (-0.64, 0.39) | 3.25 (0.04, 6.47)* | -1.1 (-5.3, 3.0) | 1.6 (-1.9, 5.0) |
| Height | -0.07 (-0.40, 0.26) | 0.45 (-0.13, 1.03) | 0.32 (-0.16, 0.81) | -0.68 (-3.68, 2.33) | 0.1 (-3.8, 4.0) | 0.0 (-3.2, 3.3) |
| **Model 3: Adjusted for maternal age, infant age, infant sex, recent illness, exclusive breastfeeding (dose-to-mother)^3^, infant weight-for-age z score** | | | | | | |
| Maternal BMI | 0.56 (-0.02, 1.13) | 0.01 (-0.99, 1.02) | 0.06 (-0.79, 0.91) | 5.08 (-0.14, 10.30) | -7.0 (-12.4, -1.6)* | -1.9 (-7.1, 3.3) |
| Percent body fat | 0.19 (-0.07, 0.46) | -0.12 (-0.58, 0.35) | -0.19 (-0.58, 0.21) | 1.78 (-0.65, 4.22) | -3.5 (-6.0, -1.1)* | -1.3 (-3.7, 1.1) |
| MUAC | 0.53 (-0.09, 1.16) | -0.04 (-1.14, 1.07) | 0.18 (-0.75, 1.12) | 4.86 (-0.87, 10.58) | -7.5 (-13.4, -1.6)* | -1.9 (-7.5, 3.8) |
| Calf circumference | 0.13 (-0.46, 0.72) | 0.51 (-0.52, 1.54) | 0.48 (-0.39, 1.35) | 1.19 (-4.21, 6.58) | -8.6 (-14.1, -3.2)* | -5.5 (-10.8, -0.3)* |
| Triceps skinfold thickness | 0.20 (-0.12, 0.51) | -0.01 (-0.57, 0.55) | -0.06 (-0.53, 0.41) | 1.80 (-1.07, 4.68) | -0.2 (-3.2, 2.8) | 1.2 (-1.6, 4.0) |
| Subscapular skinfold thickness | 0.37 (0.02, 0.72)* | 0.29 (-0.32, 0.91) | -0.12 (-0.64, 0.40) | 3.37 (0.18, 6.55)* | -2.6 (-5.9, 0.7) | 0.8 (-2.4, 3.9) |
| Height | 0.04 (-0.30, 0.38) | 0.60 (0.01, 1.20)* | 0.52 (0.01, 1.02)* | 0.38 (-2.73, 3.49) | -4.5 (-7.7, -1.3)* | -2.6 (-5.6, 0.5) |
| ^1^Note 5 participants are missing values for fat concentration/energy density, 1 participant is missing data for milk volume and 6 for energy intake.  ^2^ * p<0.05 using ANOVA for normally distributed outcome variables and Kruskal-Wallis test for non-normally distributed outcome variables  ^3^Exclusive breastfeeding defined as having less than 86.6 grams of non-milk water intake per day using the deuterium oxide dose-to-mother technique [19, 22 | | | | | | |

**Online Supporting Material**

**Supplemental Figure 2. Association of maternal BMI and percent body fat with human milk macronutrient composition and infant intake among lactating women 2 to 4 months postpartum.** Results of linear regression models where anthropometric indicators and outcome variables have been standardized; coefficients therefore represent the change in standard deviations of the outcome variable with a one standard deviation change in anthropometric indicator. Error bars show 95% confidence interval. Models are adjusted for maternal age; whether the infant was reported ill in the previous 2 weeks; child age, sex, and WAZ; and whether the infant was exclusively breastfed in the previous day.

**Online Supporting Material**

BMI

% body fat

Exclusively breastfed

Non-exclusively breastfed

▪

⬩

**Supplemental Figure 3. Association of maternal BMI and percent body fat with human milk macronutrient composition and infant intake among lactating women 2 to 4 months postpartum, stratified by exclusive breastfeeding**. Results of linear regression models where anthropometric indicators and outcome variables have been standardized; coefficients therefore represent the change in standard deviations of the outcome variable with a one standard deviation change in anthropometric indicator. Error bars show 95% confidence interval. Models are stratified by exclusive breastfeeding in the previous day, and are adjusted for maternal age; whether the infant was reported ill in the previous 2 weeks; child age, sex, and WAZ.
